# Design and Characterization of Mutated Variants of the Oncotoxic Parvoviral Protein NS1

**DOI:** 10.3390/v15010209

**Published:** 2023-01-11

**Authors:** Patrick Hauswirth, Philipp Graber, Katarzyna Buczak, Riccardo Vincenzo Mancuso, Susanne Heidi Schenk, Jürg P. F. Nüesch, Jörg Huwyler

**Affiliations:** 1 Division of Pharmaceutical Technology, Department of Pharmaceutical Sciences, University of Basel, 4056 Basel, Switzerland; 2 Proteomics Core Facility, Biozentrum, University of Basel, 4056 Basel, Switzerland; 3 Division of Clinical Pharmacology & Toxicology, University Hospital of Basel, University of Basel, 4055 Basel, Switzerland; 4 Division of Molecular Pharmacy, Department of Pharmaceutical Sciences, University of Basel, 4056 Basel, Switzerland; 5 Infection, Inflammation and Cancer Program, Division of Tumor Virology, German Cancer Research Center (DKFZ), 69120 Heidelberg, Germany

**Keywords:** H1-PV, parvovirus, infection, oncolytic virus, anticancer gene, cancer gene therapy, cancer

## Abstract

Oncotoxic proteins such as the non-structural protein 1 (NS1), a constituent of the rodent parvovirus H1 (H1-PV), offer a novel approach for treatment of tumors that are refractory to other treatments. In the present study, mutated NS1 variants were designed and tested with respect to their oncotoxic potential in human hepatocellular carcinoma cell lines. We introduced single point mutations of previously described important residues of the wild-type NS1 protein and a deletion of 114 base pairs localized within the N-terminal domain of NS1. Cell-viability screening with HepG2 and Hep3B hepatocarcinoma cells transfected with the constructed NS1-mutants led to identification of the single-amino acid NS1-mutant NS1-T585E, which led to a 30% decrease in cell viability as compared to NS1 wildtype. Using proteomics analysis, we could identify new interaction partners and signaling pathways of NS1. We could thus identify new oncotoxic NS1 variants and gain insight into the modes of action of NS1, which is exclusively toxic to human cancer cells. Our in-vitro studies provide mechanistic explanations for the observed oncolytic effects. Expression of NS1 variants had no effect on cell viability in NS1 unresponsive control HepG2 cells or primary mouse hepatocytes. The availability of new NS1 variants in combination with a better understanding of their modes of action offers new possibilities for the design of innovative cancer treatment strategies.

## 1. Introduction

Whereas most chemotherapeutic agents indiscriminately act on tumor and non-tumor tissue, oncotoxic proteins specifically interact with cancer cells only by suppressing cell growth or inducing apoptosis [1]. Oncotoxic proteins include cytokines, artificially modified secreted proteins, and proteins of viral origin, such as the parvovirus-derived oncotoxic nonstructural protein 1 (NS1) [1,2]. Usually, they are activated in tumor cells only and subsequently modulate pathways involved in cell proliferation, cell cycle control, apoptosis, mitochondrial respiration, and glycolysis [1]. Expression systems encoding for these proteins can be introduced into target cells by a viral or non-viral gene therapy approach [3,4].

The rat parvovirus H1 (H1-PV) shows low pathogenicity in normal human tissues but can infect and kill malignant cells. The natural oncotropism and oncolytic activities were therefore recently explored in phase II clinical trials [5,6,7,8]. The H1-PV viral capsid contains the linear, single-stranded DNA genome which encodes two structural and at least six nonstructural proteins, of which the 672 amino acid long NS1 is the major effector for virus propagation and cytotoxicity. The oncotoxic protein NS1 alone is sufficient to induce a strong cytotoxic effect in various human cell lines [3,4,9,10]. In a recent study, NS1 gene delivery using a non-viral vector was proposed to be a promising strategy for an anti-cancer treatment in hepatocellular carcinomas [3]. This is in contrast to viral treatment strategies, which have several drawbacks such as immunogenicity and chromosomal gene insertion [11,12].

In cancer cells, NS1 exploits and activates several distinct pathways within its host cell, not only important for virus propagation but also ultimately leading to cell cycle arrest, apoptosis, necrosis, and lysosomal cell death [2,10]. NS1 activities are modulated by post-translational protein modifications by host cell proteins. In particular, phosphorylation and acetylation of distinct NS1 amino acid residues have been shown to activate or deactivate distinct functions of the protein. In fact, side-directed mutagenesis of NS1 residues S473 and T585 to alanine, which precludes phosphorylation, resulted in a decreased effect of the protein [13,14]. Furthermore, acetylation of residues K85 and K257 of H1-PV NS1 have been shown to increase its cytotoxic effect [15].

**Figure 1:**
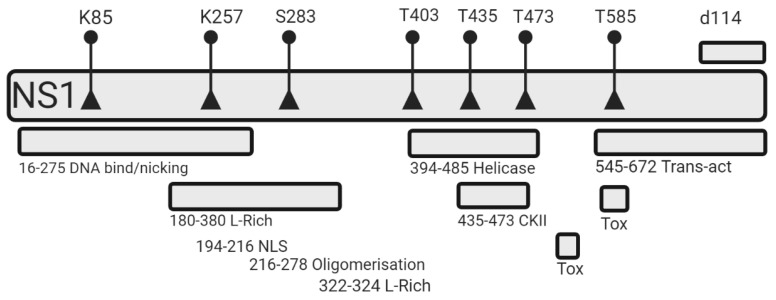
Schematic overview of non-structural protein 1 (NS1) domains and mutation sites. The non-structural parvovirus-derived protein NS1 is comprised of 672 amino acids. Amino acid positions of previously described functional domains within the NS1 protein are summarized: A site-specific DNA binding domain involved in site- and strand-specific nicking (16–275), an L-Rich area which was shown to be crucial for NS1-mediated toxicity (180–380), a nuclear localization signal (194–216), a motif controlling self-assembly into oligomers (216–278), a helicase domain including a NTP-binding pocket (394–485), a region binding CKIIα (435–473), whose interaction is needed for many NS1- signaling pathways, two toxicity domains crucial for NS1 cytotoxicity, and a transactivator domain (545–672), which positively regulates the expression of viral proteins [22]. The locations of specific acetylation [15] and phosphorylation [13,14] sites, where single amino acid mutations and a deletion of 114 nucleotides (d114) [18] were introduced during this work, are indicated. Mutations were introduced by site-directed mutagenesis. For a review see Nüesch et al. [2,10,22].

It was therefore the aim of the present study to further improve the beneficial properties (i.e., selective killing of cancer cells) of NS1 and to investigate NS1-target cell interactions. We designed single amino acid mutants of selected residues whose modification (i.e., phosphorylation or acetylation) led to altered oncotoxic NS1 activity (Figure 1). We used a phospho-mimetics approach to simulate the phosphorylated state of known serine and threonine phosphorylation sites by replacing serine or threonine to glutamic acid [16]. The acetylation of lysine was simulated by a conversion of lysine to glutamine [17]. Finally, we constructed a NS1 variant previously found in a H1-PV mutant virus, which is characterized by a 114 nucleotide in-frame deletion of NS1. This mutant virus showed increased fitness and infectivity compared to wt-H1-PV [18]. The designed mutants were analyzed with respect to their oncotoxic effects using cell viability assays, which led to the identification of an NS1 mutant (NS1-T585E) with an increased, but still specific oncotoxicity.

Previous experiments using Nanoparticle-mediated gene transfer to target hepatocellular carcinoma cells (HCC) with H-1PV NS1, have shown promising results, not only in tissue culture but in xenotransplants of nude mice as well. However, for an optimal treatment of cancers, it appears necessary to induce a bystander effect. This can be achieved through involvement of the immune-system, breaking the immune-suppressive environment and attracting immune cells through release of cytokines’ danger-associated molecular patterns (DAMPS), pathogen-associated molecular patterns (PAMPs), and, potentially, tumor-associated antigens (TAA). The latter has been shown in clinical trials with H-1PV in GBM-treated patients through peptides corresponding to NS1 and VP1, respectively [19]. Although this can be explained by a potential release through the exocytic pathway that becomes usurped in PV-infected cells to transport progeny particles to the plasma-membrane [20], it remains largely unknown whether this pathway is involved in NS1 transduced cancer cells and what kind of DAMPs or TAAs might become exposed at the surface after expression of NS1. To address these questions, the current work determines NS1-association using different proteomics approaches to identify new interaction partners of wild-type NS1 and the most potent phospho-mimetic mutant NS1-T585E of cellular proteins in NS1-transfected Hep3B cells and potential pathways triggered by NS1. Consequently, the obtained results provide significant leads to determine the potential feasibility of an NS1-associated treatment of cancers involving immune-attraction and to obtain a bystander effect.

Although, H-1PV has shown proof-of-concept in a phase I/IIa clinical trial, there are still limitations regarding susceptibility of cancer cells/entities to this virus species. This can, for instance, be overcome through nanoparticle-associated transfer of the cytotoxic NS1 protein and/or NS1-encoding nucleic acids either alone or in combination with other (viral) proteins [10,21]. In addition, there is a large potential to improve efficacy, particularly through engagement of the host immune system in order to induce an anti-tumor immune response through a combination of danger-associated molecular patterns (DAMPs) and pathogen-associated molecular patterns (PAMPs) released from infected/transfected cancer cells. The current manuscript aims to use a virus-free approach to determine the impact of NS1 on the host cell using wildtype and site-directed mutant H1-PV NS1 to identify new cellular targets and interaction partners of NS1. This includes well-established targets necessary for viral replication, such as factors involved in DNA replication and repair, exocytosis, and potential danger-associated molecular patterns associated with cytolysis.

## 2. Materials and Methods

### 2.1. Materials

All chemicals were purchased from Sigma Aldrich (Buchs, Switzerland) and were of analytical grade. Strepatavidin Sepharose High Performance beads were purchased from GE Healthcare (Chicago, IL, USA). cOmplete Protease Inhibitor Cocktail was obtained from Roche (Basel, Switzerland). Dulbecco’s phosphate buffered saline (DPBS, without calcium and magnesium), Dulbecco’s modified Eagle medium (DMEM, high glucose), 0.25% Trypsin/EDTA, 100 × Penicillin/Streptomycin solution, and poly-D-lysine were obtained from Sigma Aldrich (Buchs, Switzerland). OptiMEM (Gibco) and Lipofectamine 3000 (Invitrogen, Waltham, MA, USA) were obtained from Fisher Scientific AG (Reinach, Switzerland), and foetal calf serum was purchased from Amimed (Bio-concept, Allschwil, Switzerland). Tissue culture plates were purchased from TTP (Trasadingen, Switzerland). Hep3B and HepG2 cells were obtained from ATCC (Manassas, VA, USA). Restriction endonucleases, DNA ligases, and polymerases were obtained from New England Biolabs Inc. (Ipswich, MA, USA). QIAquick PCR Purification Kit, QIAprep Spin Miniprep Kit, and QIAprep Plus Midiprep Kit were purchased from Qiagen (Hilden, Germany). pcDNA3.1+ (Invitrogen) was purchased from Fisher Scientific AG (Reinach, Switzerland), pTagGFP-N was purchased from Evrogen (Moscow, Russia) and MCS-BioID2-HA was purchased from Addgene (Watertown, MA, USA). E. coli DH5alpha (Invitrogen) were obtained from Fisher Scientific AG (Reinach, Switzerland).

### 2.2. Cloning of NS1 for Cytotoxicity Studies

Specific NS1-mutants were created by overlap extension PCR and cloned into pcDNA3.1+. pcDNA3.1-NS1 was used as a template for the first round of PCRs. Briefly, two separate overlapping PCR fragments were created using the flanking primers p1 and p2 in combination with the respective mutated reverse or forward primers (see Table S1). The resulting two overlapping PCR products were purified (QIAquick PCR Purification Kit) and subsequently combined at equimolar concentrations to perform an overlap extension PCR, using the outermost primers (p1, p2) to recombine the two mutated fragments. All PCR reactions were carried out using Phusion High-Fidelity DNA Polymerase (New England Biolabs) according to the manufacturer’s recommendations. DNA was initially denatured at 98 °C for 30 s followed by 35 cycles of denaturation (98 °C, 10 s), annealing (68 °C, 30 s), and elongation (72 °C, 45 s). Final extension was carried out at 72 °C for 10 min. The final PCR products were digested and ligated into pcDNA3.1+ using BamHI and NotI restriction sites.

### 2.3. Cloning of Plasmids for Proteomics

For GFP pull down studies, NS1-wt and NS1(T585E) were cloned into pTag-GFP-N Evrogen or pTag-NS1-GFP [3]. pTag-NS1(T585E)-GFP was created by PCR using pcDNA3.1-NS1(T585E) as a template and the flanking primers p1 and p3 as described above. The resulting PCR product was digested and ligated into pTag-GFP using BamHI restriction sites.

For proximity-dependent biotinylation analysis, NS1-wt and NS1(T585E) were amplified by PCR using the respective pcDNA3.1-construct as template and the flanking primers p4, p5. The PCR products were digested and ligated into the MCS-BioID2-HA (Addgene) plasmid using AgeI sites. MCS-BioID2-HA control plasmid was modified to introduce a start codon in the BioID2-gene. Therefore, the original MCS-BioID2-HA plasmid was used as a template and amplified by PCR using primers p22 and p23 as described above. The PCR product (ATG-BioID2-HA) was digested with AgeI and HindIII and ligated into MCS-BioID2-HA, of which the original BioID2 sequence was removed by digestion with the same restriction enzymes.

### 2.4. Sequencing and Amplification of Plasmid DNA for Transfections

All plasmids were transformed into the chemically competent *E. coli* DH5alpha strain. pDNA from individual colonies was isolated using QIAprepSpin miniprep kit and sequenced to confirm correct sequence and reading frame of the insert. Plasmids that were used for further experiments were purified using the QIAprep Plasmid PlusMidi Kit (Qiagen) in accordance with the manufacturer’s recommendations. Plasmids used in the present study are listed in Table 1.

### 2.5. Cell Culture and Transfection

Hepatocarcinoma cell lines HepG2 and Hep3B were maintained in DMEM high glucose (4.5 g/L), supplemented with 10% fetal calf serum and Penicillin-Streptomycin, referred to as complete culture medium (CCM). Cells were cultured at 37 °C in a humidified CO_2_-incubator (5% CO_2_). Sub-cultivation was performed twice a week and the cells were kept in culture between passage numbers 22 to 40.

For transfection, cells were seeded at indicated cell densities on poly-D-Lysine (3 μg/cm^2^) coated tissue culture plates. Twenty-four hours after seeding, cells were transfected with respective plasmid DNA (pDNA) using Lipofectamine 3000 (Invitrogen) at 2:1 *v*/*w* ratio of plasmid DNA to Lipofectamine 3000. In brief, pDNA was diluted in OptiMEM (Gibco) and mixed with P3000 reagent. Lipofectamine 3000 was diluted with OptiMEM, mixed with the pDNA-P3000-mixture, vortexed, and incubated for 5 min at room temperature before adding dropwise to the cells. To reduce toxic effects of the transfection reagent, the medium was changed 12 h after transfection.

### 2.6. MTT Cell Viability Assay

In-vitro cell viability was tested using the MTT assay as described [23]. In brief, 8000 cells per well were seeded in 200 μL of CCM into poly-D-lysine coated 96-well plates. Twenty-four hours after seeding, medium was reduced to 150 μL/well, and the cells were transfected with 0.1 μg of pDNA3.1-NS1-constructs as described above. Seventy-two hours post-transfection, the culture medium was reduced to 50 μL per well, and 50 μL of MTT working solution (1 mg/mL) was added. Cells were incubated for 2 h at 37 °C. Formazan crystals were dissolved with DMSO on a shaker in the dark for 30 min at RT. Absorption was measured at 540 nm. Mock transfected cells (i.e., transfections with empty pcDNA3.1+) were used as 100% reference of cell viability.

### 2.7. Sample Preparation for Phospho- and Total Cell Proteomics

Cells (2 × 10^6^ cells) were analyzed 48 h post-transfection. Two mililiters ice-cold DPBS containing cOmplete Protease Inhibitor Cocktail (Roche) was added and cells were harvested on ice with a cell scraper. Cells were collected by centrifugation (115 min at 300× *g*, 4 °C). Washed cells were snap-frozen in liquid nitrogen and stored at −80 °C until use. Cells were lysed in 8 M Urea (Sigma) and 0.1 M ammonium bicarbonate in the presence of phosphatase inhibitors (Sigma) using ultra-sonication (Bioruptor, Diagenode, Belgium). Protein concentration was determined by the BCA assay (Thermo Fisher Scientific, Waltham, MA, USA). Two hundred μg protein was reduced with 5 mM TCEP for 60 min at 37 °C and alkylated with 10 mM chloroacetamide for 30 min at 37 °C. After dilution with 100 mM ammonium bicarbonate buffer to a final urea concentration of 1.6 M, proteins were digested by incubation with sequencing-grade modified trypsin (Promega, Madison, WI, USA) overnight at 37 °C. After acidification using 5% TFA, peptides were desalted on C18 reversed-phase spin columns (Macrospin, Harvard Apparatus, Holliston, MA, USA) and dried under vacuum.

Peptide samples were enriched for phosphorylated peptides using Fe(III)-IMAC cartridges on an AssayMAP Bravo platform as described above [24]. Remaining flow-through fractions of non-phosphorylated peptides were labeled with tandem mass isobaric tags (TMT 16-plex, Thermo Fisher Scientific). Desalted TMT-labeled peptides were fractionated by high-pH reversed phase separation using a XBridge Peptide BEH C18 column (3.5 µm, 130 Å, 1 mm × 150 mm, Waters) on an Agilent 1260 Infinity HPLC system, as described previously [25].

### 2.8. Sample Preparations for (NS1-)GFP-Pulldowns

GFP pulldown was performed using a GFP-Trap Magnetic Agarose Kit (chromotek). Briefly, cell pellets were resuspended in an ice-cold 10 mM Tris/Cl pH 7.5, 150 mM NaCl, 0.5 mM EDTA, 0.5%, Nonidet P40 Substitute, 0.09% sodium azide, cOmplete protease inhibitor cocktail (Roche). Samples were centrifuged at 17,000× *g* for 10 min at 4 °C, diluted with 10 mM Tris/Cl pH 7.5, 150 mM NaCl, 0.5 mM EDTA, 0.018% sodium azide) supplemented with 1 mM PMSF and protease inhibitor cocktail, and separated from the supernatant with a magnet. Proteins were eluted by on-bead digestion in 1,6 M Urea, 100 mM Ammonium bicarbonate, 5 μg/mL trypsin, pH 8 for 30 min at 27°, followed by reduction in 1.6 M Urea, 100 mM Ammonium bicarbonate, and 1 mM TCEP, pH 8. Reduced sulfhydryl groups were alkylated using chloroacetamide (15 mM) prior to fragmentation of peptides by a second tryptic digest (12 h at 37 °C). The tryptic digest was acidified (pH < 3) using TFA, desalted using C18 reversed phase spin columns (Microspin, Harvard Apparatus), and subjected to LC-MS analysis as described above.

### 2.9. Sample Preparations for BioID2

BioID protein-proximity labeling in living Hep3B cells was done as described previously [26]. Ten hours after transfection, biotin was added to cells using a final concentration of 50 μM. Forty-eight hours post-transfection, cells were harvested, washed, and lysed. Biotinylated peptides were isolated using streptavidin-Sepharose beads equilibrated in lysis buffer. On-bead digestion was performed as described in the previous section. Peptides were purified and subjected to LC-MS analysis as described above.

### 2.10. MS Data Acquisition

Phosphorylated peptides were resuspended in 0.1% formic acid and analyzed using Orbitrap Fusion Lumos Mass Spectrometer fitted with an EASY-nLC 1200 (both Thermo Fisher Scientific) and a custom-made column heater set to 60 °C. Peptides were resolved using a RP-HPLC column (75 μm × 36 cm) packed in-house with C18 resin (ReproSil-Pur C18–AQ, 1.9 μm resin; Dr. Maisch GmbH) at a flow rate of 0.2 μL/min. The MS was operated in DDA mode with a cycle time of 3 s. MS1 scans were acquired at a resolution of 120,000 FWHM (at 200 *m*/*z*) and a scan range from 375 to 1600 *m*/*z*. Precursors were isolated with the isolation window of 1.4 *m*/*z* and fragmented with HCD with CE set to 30%. MS2 scans were acquired at a resolution of 30,000 FWHM. Both scans were acquired using Orbitrap.

TMT fractions were resuspended in 0.1% formic acid and analysed using a Q Exactive HF Mass Spectrometer fitted with an EASY-nLC 1000 (both Thermo Fisher Scientific). Column parameters were as above except in length (30 cm). The MS was operated in Top10 DDA mode. MS1 scans were acquired at a resolution of 120,000 FWHM (at 200 *m*/*z*) and a scan range from 350 to 1600 *m*/*z*. Precursors were isolated with the isolation window of 1.1 *m*/*z* and fragmented with HCD with CE set to 30%. MS2 scans were acquired at a resolution of 30,000 FWHM.

Peptides derived from GFP-pull-down and BioID experiment were resuspended in 0.1% formic acid and analysed using LTQ-Orbitrap Elite Mass Spectrometer fitted with an EASY-nLC 1000 (both Thermo Fisher Scientific). Column parameters were as above. The MS was operated in Top20 DDA mode. MS1 scans were acquired using Orbitrap at a resolution of 120,000 FWHM (at 400 *m*/*z*). The 20 most abundant precursors were CID fragmented (CE 35%) and analysed in linear ion trap.

### 2.11. MS Data Analysis

The acquired raw LFQ data-files (PO4 and GFP/BioID) were processed using Progenesis QI software (v2.0, Nonlinear Dynamics Limited) to extract peptide precursor ion intensities. Results were searched against the human proteome database (UNIPROT) using MASCOT, following criteria:mass tolerance of 10 ppm (precursor) and 0.02 Da (fragments)/0.6 Da (fragments) for orbitrap and for ion trap MS2 data, respectively, full tryptic specificity, 3 missed cleavages allowed, carbamidomethylation (C) set as fixed modification and oxidation (M) set as variable modification.

The TMT raw files were searched against human proteome database (UNIPROT) using SpectroMine software (Biognosys). Standard Pulsar search settings for TMTpro were used. Raw reporters ions intensities were exported for further analysis.

Quantitative analysis results from both label-free and TMT quantification were processed using the SafeQuant R package v. 2.3.2. [25], to obtain peptide relative abundances.

### 2.12. Statistical Analysis

Values are provided as means ± SEM of the indicated number of experiments comprising each *n* ≥ 3 measurement. Statistically significant differences in viability between transfected and control cells were identified by ANOVA followed by Student’s *t*-test and Bonferroni correction for multiple comparisons. Statistical analysis was done using OriginPro 2018 software (OriginLab, Northampton, MA, USA). The level of significance: 0.05. Proteomics analysis included adjustment of reporter ion intensities, global data normalization by equalizing the total reporter ion intensity across all channels, summation of reporter ion intensities per protein and channel, calculation of protein abundance ratios, and testing for differential abundance using empirical Bayes moderated t-statistics. Calculated q-values were corrected for multiple testing using the Benjamini−Hochberg method. Proteins with a significantly higher abundance in NS1-wt and NS1-T585E compared to control pulldowns, respectively, (q < 0.05) were analyzed with Gene Ontology enRIchment anaLysis and visuaLizAtion (GORILLA) tool [27] (background based analysis, background dataset (from uniprot.org), and target datasets were provided at https://doi.org/10.5281/zenodo.6423418), to identify biological processes and cellular compartments, which are affected based on these proteins. Raw data files and statistical values used for analysis are provided in the repository zenodo.org (https://doi.org/10.5281/zenodo.6423418). Data are also available via ProteomeXchange with identifier PXD036350.

## 3. Results

### 3.1. Effect of NS1 Mutations on Cytotoxicity

**Figure 2:**
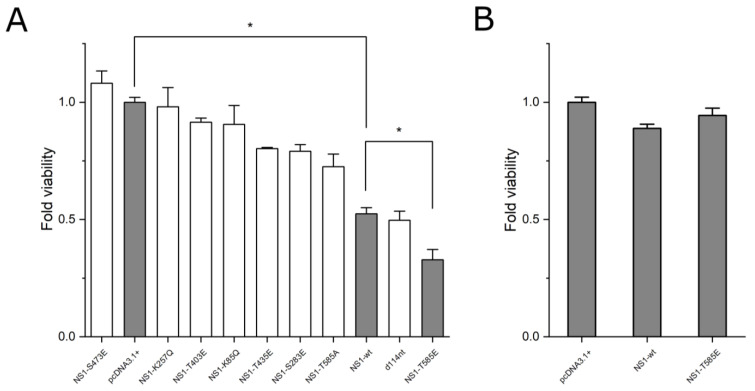
Cell toxicity assays; MTT assays of cells transfected with empty pcDNA3.1+ vector (mock), pcDNA3.1-NS1-wt, or with pcDNA3.1-NS1-mutants. Relative viability was determined 72 h post-transfection and normalized to pcDNA3.1+ (mock) transfection; (**A**) MTT assay using NS1-responsive Hep3B cells; relative viabilities of NS1-wt and NS1-T585E expressing cells are highlighted in grey; (**B**) MTT assay using NS1-unresponsive HepG2 cells (control). Means +/− SEM (*n* ≥ 5). *: *p* ≤ 0.05. Grey bars: 100% reference, NS1-wt reference, and most potent mutant, respectively.

We mutated seven selected amino acid residues within NS1 whose modification (i.e., phosphorylation or acetylation) was suggested to lead to altered oncotoxicity. Lysine residues 85 and 257, whose acetylation had been suggested to increase their oncotoxic effects were mutated to glutamine to mimic their acetylated state. Serine residues 283 and 473 and threonine residues 403, 405, and 585 were mutated to glutamic acid to mimic their phosphorylated state. Additionally, we constructed a NS1 variant previously found in a H1-PV mutant virus, which is characterized by a 114 nucleotide in-frame deletion of NS1-wt residues 1760 to 1873. NS1-wt responsive Hep3B cells were transfected and their effect on cell viability was assessed using the MTT assay 72 h post-transfection. Transfection efficiency was reproducibly shown to be 54%; SD = 0.98% (*n* = 3) after pTag-GFP transfection. Therefore, viability was corrected by 0.5 to compensate for non-transfected cells. Lysine mutants K85Q and K257Q, as well as serine mutation S473E led to a loss of the cytotoxic effect of NS1 and a cell viability not significantly different to mock transfection (*p* > 0.05). NS1 mutants T403E, T435E and S283E showed a significantly lower cytotoxic effect compared to NS1-wt, but a significantly higher cytotoxic effect compared to mock transfection (*p* < 0.05). The 114nt deletion variant (NS1-del-114) led to no significant difference in cell viability compared to NS1-wt in Hep3B cells (*p* > 0.05). However, the NS1 mutant T585E led to a 30% decrease of cell-viability compared to NS1-wt and 63% decrease of cell viability compared to mock-transfection in Hep3B cells (*p* < 0.05). To confirm that the increased cytotoxic effect of NS1-T585E was due to the mutation of threonine 585 to the phosphor-mimetic glutamic acid, the same residue was mutated to alanine (NS1-T585A), which resulted in a lower oncotoxic effect compared to NS1-wt (*p* > 0.05) (Figure 2A). Of note, neither NS1-wt nor NS1-T585E overexpression led to a decrease in cell viability in NS1 unresponsive control HepG2 cells (Figure 2B) or primary mouse hepatocytes (Supplement Figure S3).

The increment of mitochondrial reactive oxygen species (ROS) levels within cells after NS1-wt transfection has previously been described [9]. To assess if the elevated cytotoxic effect of NS1-T585E compared to NS1-wt was linked to elevated ROS levels, we transfected NS1-responsive Hep3B cells with either pTag-NS1-wt-GFP, pTag-NS1-T585E-GFP or pTag-GFP-N (control) and compared ROS levels of successfully transfected cells using the MitoSOX assay and FACS analysis. Indeed, Hep3B cells overexpressing NS1-T585E-GFP and NS1-wt-GFP showed a higher ROS level compared to GFP only control cells (Supplement Figure S1). Control experiments with NS1-unresponsive HepG2 cells resulted in no elevated ROS levels (Supplement Figure S2).

### 3.2. Proteomics: Effects of NS1-wt and NS1-T585E on the Host-Cell Proteome

**Figure 3:**
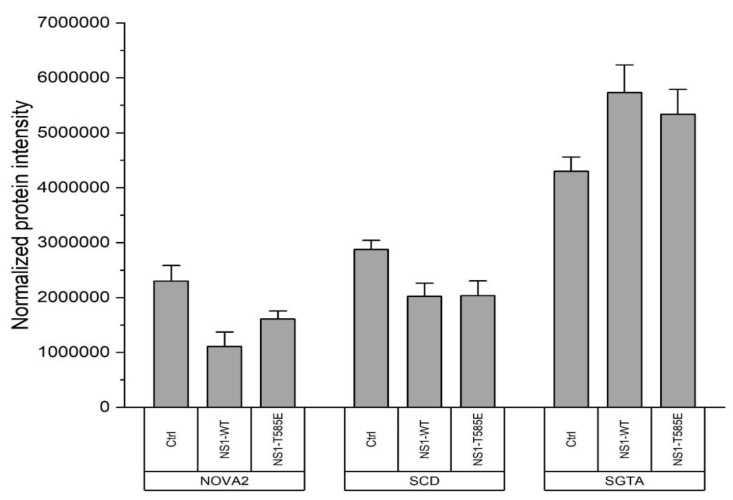
Expression levels of selected proteins after NS1-wt and NS1(T585E) transfection. Hep3B-cells were transfected with pcDNA3.1-NS1-wt, pcDNA3.1-NS1(T585E), or pcDNA3.1+. Normalized protein intensities identified by mass spectrometry for proteins with reproducibly altered expression levels (NOVA2, SDC and SGTA) are shown. Values are means ± SEM, *n* = 4.

Different proteomic approaches were used to further elucidate and compare the interactions of NS1-wt and mutant NS1-T585E with the host cell proteome, their effects on host-cell protein expression levels, and phosphorylation patterns in NS1-responsive cells. In a first set of experiments, Hep3B cells were transfected with either pcDNA3.1-NS1-wt, pcDNA3.1-NS1-T585E or pcDNA3.1+ (mock transfection). Forty-eight hours post-transfection, the cell proteome was analyzed using mass spectrometry. Three proteins showed a consistently altered expression level across all replicates (q < 0.05) compared to mock transfection in both NS1-wt and NS1-T585E overexpressing cells. NOVA Alternative Splicing Regulator 2 (NOVA2) and Stearoyl-CoA Desaturase (SCD) were downregulated. Enhanced expression was observed for Small Glutamine Rich Tetratricopeptide Repeat Co-Chaperone Alpha (SGTA) (Figure 3). A list of all differentially regulated genes identified during this study can be found at the repositories zenodo.org (https://doi.org/10.5281/zenodo.6423418) and ProteomeXchange with identifier PXD036350. A volcano plot depicting the changes in the proteome after NS1-wt and NS1-T585E can be found in the supplement (Supplement Figure S4).

### 3.3. Proteomics: Analysis of Phosphorylation Patterns

Hep3B cells were transfected with either pcDNA3.1-NS1-wt, pcDNA3.1-NS1-T585E or pcDNA3.1+ (mock transfection). Forty-eight hours post transfection, proteins of cell lysates were digested; phosphopeptides were enriched and subsequently quantified by mass spectrometry. NS1-wt overexpression led to a significantly altered (q < 0.05) phosphorylation pattern of four proteins: Apoptotic Chromatin Condensation Inducer 1 (ACIN1), Small Glutamine Rich Tetratricopeptide Repeat Co-Chaperone Alpha (SGTA), HIRA Interacting Protein 3 (HIRIP3), and AMMECR Nuclear Protein 1 (AMMECR1).

NS1-T585E overexpression did not lead to any statistically significant differences in phosphorylation patterns compared to mock-plasmid transfection. However, there was a trend towards a higher phosphorylation of ACIN1 at position T254 in cells expressing NS1-T585E. A list of all proteins identified during the phosphor-enrichment experiments can be found at the repositories zenodo.org (https://doi.org/10.5281/zenodo.6423418) and ProteomeXchange with identifier PXD036350. A volcano plot depicting the changes in the phosphorylation patterns after NS1-wt and NS1-T585E can be found in the supplement (Supplement Figure S5).

### 3.4. Proteomics: Binding of or Close Proximity to NS1

Hep3B-cells were transfected with pTag-NS1-wt-GFP, pTag-NS1-T585E-GFP, or pTag-GFP-N (control-transfection). GFP or NS1-GFP fusion proteins were pulled down with a GFP-Antibody. Subsequently, cellular proteins binding directly or indirectly to NS1 were identified by mass spectrometry. Proteins with a significantly (q < 0.05) higher abundance in NS1-wt-GFP and NS1-T585E-GFP compared to GFP-only pulldowns, respectively, were grouped according to selected biological processes and cellular compartments associated with viral infection, stress responses, regulation of gene expression, regulation of the immune system, regulation of the cell cycle, the cytoskeleton, sites of DNA damage, mitochondria, and the ribonucleoprotein complex.

**Figure 4:**
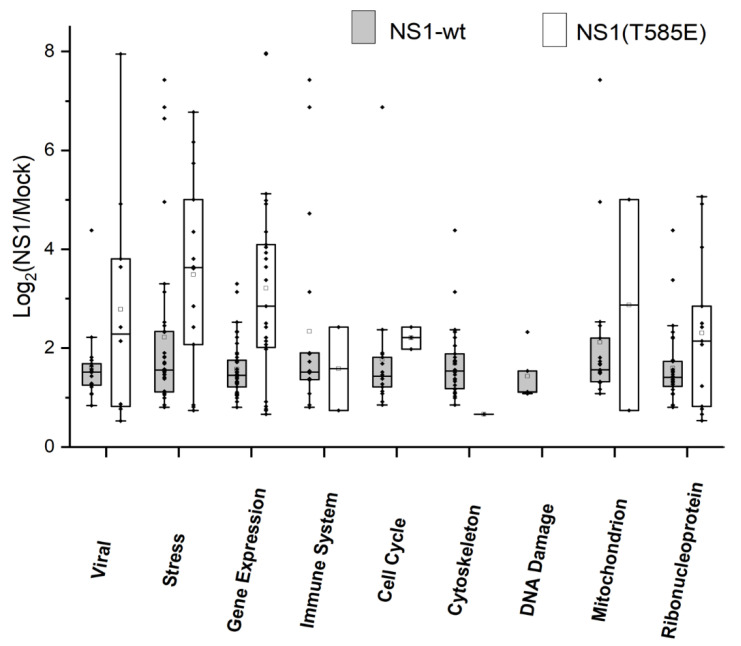
Identification of binding partners of NS1-wt and NS1-T585E by proteomics. Hep3B-cells were transfected with pTag-NS1-wt-GFP, pTag-NS1-T585E-GFP, or pTag-GFP-N. NS1-GFP fusion proteins were pulled down and cellular proteins binding to NS1 were identified by mass spectrometry. Proteins with a significantly higher abundance in NS1-wt-GFP or NS1-T585E-GFP compared to GFP-only pulldown, respectively, are grouped with respect to the indicated biological processes or cellular components. Box plot representation of median, 1st and 3rd quartile, whiskers as quartiles plus 1.5 times the interquartile range (IRQ), and outliers.

We identified 120 proteins that were significantly enriched using the NS1-wt-GFP approach and 51 using the NS1-T585E-GFP approach. Of these proteins, 16 were significantly enriched in both NS1-wt-GFP and NS1-T585E-GFP pull-downs. The log2 ratios intensities of NS1-wt-GFP/mock or NS1-T585E-GFP/mock of the identified proteins associated with the analyzed biological processes and/or cellular components are shown in Figure 4.

**Figure 5:**
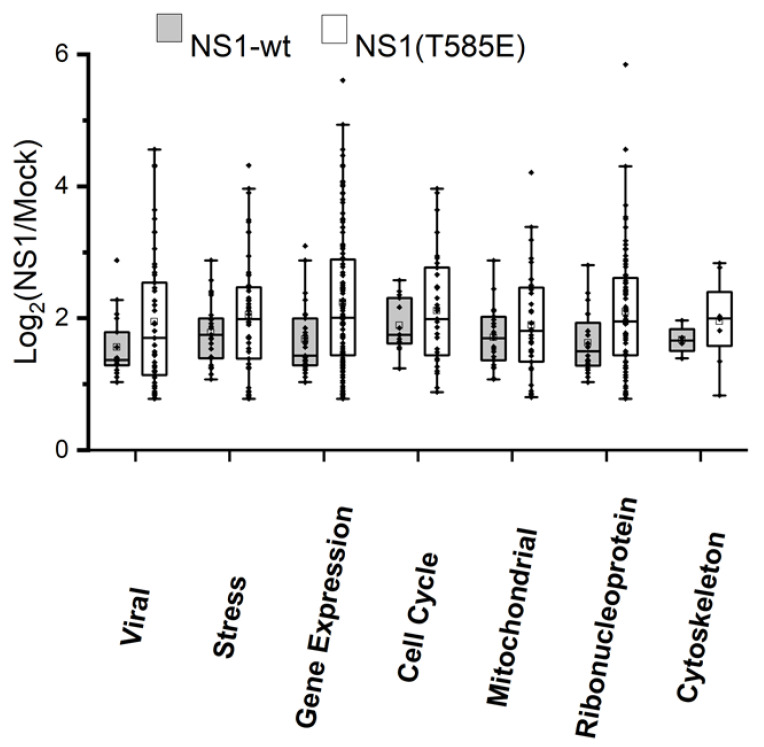
Proteomics-based identification of proteins within close proximity to NS1-wt and NS1-T585E. Hep3B cells were transfected with NS1-wt-BioID2 or NS1-T585E-BioID2 or BioID2 only. Proteins in close proximity and therefore biotinylated by BioID2 were pulled down using streptavidin beads. Proteins identified by mass spectrometry with a significantly higher abundance compared to BioID2-only control are grouped with respect to the indicated biological processes or cellular components. Box plot representation of median, 1st and 3rd quartile, whiskers as quartiles plus 1.5 times the interquartile range (IRQ), and outliers.

In order to identify additional proteins that potentially interact with NS1 or NS1-T585E, we used a proximity-dependent biotinylation technology. Thereby, NS1-wt or NS1-T585E were fused to the N-terminus of the promiscuous biotin ligase BioID2. Hep3B-cells were transfected with pNS1-wt-BioID2-HA, pNS1-T585E-BioID2-HA, or pBioID2-HA (mock transfection). Proteins in close proximity to the biotin ligase and thus to NS1-wt or NS1-T585E were biotinylated, pulled down with streptavidin beads and subsequently identified by mass spectrometry. Proteins with a significantly (q < 0.05) higher abundance of NS1-wt-BioID2-HA and NS1-T585E-BioID2-HA compared to GFP-only pulldowns, respectively, were further analyzed using gene ontology enrichment analysis [27] to identify biological processes and cellular compartments, which are associated with the pulled down proteins. As described above, results were grouped. We identified 92 proteins which were significantly enriched using the NS1-wt-BioID2 approach and 267 proteins using the NS1-T585E-BioID2 approach. Results are summarized in Figure 5.

Individual proteins identified in both the GFP-trap and the BioID2 approach can be listed as follows: Of the 120 proteins identified in the NS1-wt-GFP pull-down and 92 proteins of the NS1-wt-BioID2 approach, seven proteins were identified by both approaches. These were LIG3, RPL11, HSD17B10, MCM4, RPL9, TUBA1C, and TUBB. Similarly, of the 51 proteins identified in the NS1-T585E-GFP pull-down and 267 proteins of the NS1-T585E-BioID2 approach, 15 proteins were identified by both approaches. These were LIG3, RPL11, BANF1, DDX39A, HIST1H4A, HNRNPU, HSPA9, PRDX1, RBMX, RFC2, RFC4, RNPS1, RPS4X, SRP14, and TRA2B. Of note, only LIG3 and RPL11 were identified in all four experiments. Tubulin seems to be in close proximity to both NS1-wt and NS1-T585E. However, it is only bound to NS1-wt.

## 4. Discussion

### 4.1. NS1 Mutant Design

The aim of the present study was to improve the oncotoxic properties of NS1 in view of its potential use in cancer therapy and to study NS1-target cell interactions. We therefore designed eight single amino acid mutants by exchanging NS1 residues, which were previously described as post-translationally modified and thereby as regulating NS1 cancer cell specific NS1 cytotoxicity [13,14,28,29]. Instead of mutating phosphorylation sites (serine or threonine residues) to alanine, as described before [13,14,28,29], we used a phosphomimetic approach, which included the mutation of serine or threonine residues to glutamic acid. The charge and size of the glutamic acid side chain resembles the properties of phosphorylated serine or threonine side chains and may therefore mimic the functional properties of the phosphorylated state of a protein. The validity of this approach to studying the effects of phosphorylated residues on protein functionality has been described in several studies [30,31,32]. In this study, therefore, we exchanged NS1 residues S283, T403, T435, S473 and T585 with glutamic acid. Acetylation of K85 and K257 has previously been shown to enhance the cytotoxic effect of NS1 [15]. In addition to phosphomimetic mutations, we substituted lysine residues K85 and K257 for glutamine (Q) to simulate an acetylated state of these residues. This acetylation mimicry has also been described previously [33,34,35]. Last, we introduced a 144nt in-frame deletion to NS1, which has been proposed as leading to an increased viral fitness of H1-PV (i.e., infectivity, viral spread, number of progenitors) in human newborn kidney (NB-324K) cells [18,36].

### 4.2. Cell Viability Assays

The cytotoxic effect of NS1 is dependent on highly specific host-cell activation and in human cells, therefore, is limited to certain types of cancer cells [3,37]. To investigate if any of the designed mutants has an increased cytotoxic effect in NS1 responding cancer cells, we conducted cell viability assays in NS1 responsive Hep3B hepatocellular carcinoma cells, using unresponsive HepG2 cells as control. NS1 mutant T585E expression led to a 63% lower cell viability compared to the control (mock-transfection) and 30% lower cell-viability as compared to NS1-wt in Hep3B cells. This finding is consistent with the observation that a threonine to alanine mutation of this residue leads to impaired NS1 functionality [13]. It can be concluded that T585 phosphorylation, which occurs at a late stage in the viral life-cycle, is crucial for the cytotoxic functions of NS1 [13]. Indeed, our data confirms a decreased cytotoxicity of NS1-T585A compared to NS1-wt (Figure 2A). The 114nt- deletion in-frame H1-PV mutant, which, it is suggested, leads to an increased viral fitness and an enhanced capability to suppress tumor growth [18,36], showed a cytotoxicity comparable to NS1-wt in our hands. This might indicate that for a beneficial effect of the deletion, an interplay between NS1-d114nt and other viral proteins is needed. The effect of the T585E mutation within the viral genome in regards to the cancer-specific effects of the whole virus should be explored in future studies. Interestingly, all other mutants designed in this study showed, compared to NS1-wt, a decreased cytotoxicity (NS1-S283E and NS1-T435E) or a complete loss of the cytotoxic effect (K85Q, K257Q, T403E, S473E). The impaired functionality of proteins bearing phosphorylation- or acetylation-mimicking mutations might have several reasons. Glutamic acid, for example, does not have the hydration layer and formal charges of phosphate, the mutations might lead to structural alternations of the protein which might affect protein functionality, or phosphor-mimetic mutants might lose a potential ability to act as a kinase [38,39,40]. Of note, observed cytotoxic effects were paralleled by an increase in reactive oxygen species (ROS). Note that that the mutation of NS1 at residue T585 did not impair its expression. A similar NS1 mutant (S585A) has been introduced into MVMp leading to viruses with reduced toxicity [13]. In addition, NS1-T585E expression was demonstrated by proteomics analysis (see Data at repository (https://doi.org/10.5281/zenodo.6423418)). Analysis of protein expression by proteomics was limited to NS1-T585E because non-active NS1 mutants were of no interest in the present project and were therefore not further investigated.

Importantly, the increased cytotoxic effect of NS1-T585E on NS1-responsive Hep3B cells could not be observed in NS1-unresponsive HepG2 cells or primary mouse hepatocytes, and the associated increase in ROS could also not be observed in HepG2 cells. As described before, NS1-wt is toxic only in certain types of cancers, depending on pathways often activated in transformed cells, whereas other cell types, amongst them untransformed, healthy cells, are unaffected by NS1 cytotoxicity [3]. The same accounts for the whole H1-parvovirus [4,41], which led to the investigation of the virus in phase II clinical studies [8]. It is therefore crucial for any possible application as an anticancer treatment strategy to maintain the specificity of the cytotoxic effect of NS1-mutants. Cytotoxic effects should be limited to certain cancer cell types while non-diseased cells should not be affected [1].

### 4.3. Induced Changes in the Host-Cell Proteome afterNS1 Expression

A comprehensive study to decipher the interactome of the multifunctional parvoviral non-structural protein NS1 is prone to difficulties, particularly in a cell-free environment. Indeed, to be able to interact directly with several components of a multiprotein-complex, such as the replication machinery or DNA-repair complex, NS1 needs to oligomerize, a process that is dependent on interaction with ATP (or non-hydrolysable ATPs), causing essential conformational alterations [14,28,42]. Therefore, methods to mimic dimerization, such as an N-terminal GST-tag or crosslinking using antibodies [43,44,45], proved helpful to identify NS1-interaction partners from cellular extracts and to show NS1 interaction with DNA-recognition elements, respectively. Alternatively, proximity-ligation assays could verify the presence of NS1 in the close vicinity of partner proteins in the context of (living) cells.

To get a better understanding on how NS1 exerts its effects on the host cell and why mutant NS1-T585E has an increased effect compared to NS1-wt, we used different proteomic approaches to detect changes in the host cell proteome, including phosphorylation patterns. Furthermore, we identified direct or indirect interaction partners and proteins in close proximity to NS1. On the proteome level, NS1-wt and NS1-T585E expression led to a detectable change of the expression level of only three proteins after 48 h. First, downregulation was observed for NOVA Alternative Splicing Regulator 2 (NOVA2), which is an alternative splicing factor, proposed to also be involved in tumor progression [46]. The extensive involvement of NS1 in the host-cell gene transcription machinery has been previously described [2]. Interestingly, previous work suggests a role for NOVA2, which is upregulated in a variety of cancer cell types, in Wnt signaling. NOVA2 has been proposed to be a positive regulator of β-catenin [47], which, in turn, promotes the expression of a variety of oncogenes, therefore promoting transformation and tumor progression [48]. A downregulation of NOVA2 by NS1 could therefore be a further explanation for the suppression of tumor growth upon NS1 expression. Stearoyl-CoA Desaturase (SCD) is another protein that shows decreased expression levels upon NS1 expression (*wt* and *mutant*). It has been shown that higher SCD levels in cancer cells lead to resistance to ROS induced apoptosis and downregulation of SCD rendered cancer cells that are more sensitive to apoptosis [49]. SCD is overexpressed in different cell lines including liver cancer cell lines such as Hep3B [50].SCD utilizes cytochrome *b*5 electrons and oxygen for fatty acid biosynthesis and is linked to insensitivity to ROS induced apoptosis in certain cancers [50,51,52,53]. We speculate that NS1 mediated downregulation of SCD renders Hep3B cells more sensitive to ROS and drug induced apoptosis [49,50], offering an additional explanation of how NS1 exerts its cytotoxic effects. Upregulation was observed for Small Glutamine Rich Tetratricopeptide Repeat Co-Chaperone Alpha (SGTA), which has been previously proposed to interact with and be modified by NS1 of H1-PV [54]. The interaction of NS1 with SGTA is further supported by our observation that SGTA is upregulated upon NS1 (*wt* and *mutant*) expression in Hep3B cells. SGTA has also been shown to be involved in various viral pathways and in protein quality control and localization [55], which is consistent with the extensive involvement of NS1 in the host-cell expression machinery previously described [2].

In the NS1-GFP pull-down experiment, we identified more interaction partners binding to NS1-wt than to the mutant NS1-T585E. This could be explained by a more rigid, less flexible conformation of the mutant NS1-T585E compared to NS1-wt. The latter can adapt its conformation depending on the phosphorylation state of residue T585 and thus interact with a broader variety of proteins. On the other hand, the reduced flexibility of NS1-T585E allows for a more specific and stronger interaction with a given binding partner. It should be noted that the functionality of the NS1-GFP fusion protein was demonstrated and confirmed previously [3].

Because the GFP pull-down reveals only proteins that are tightly associated with NS1, other interaction partners that bind only loosely or in an on/off manner might be lost. Therefore, we used the BioID2 approach to identify proteins in close proximity to NS1-wt or NS1-T585E. Interestingly, in case of the proximity study, not only a larger variety of different proteins but also often a larger ratio of signal intensities of the individual proteins was identified with NS1-T585E compared to NS1-wt. We speculate that the fewer but more stable interactions of NS1-T585E with cellular proteins (as shown in GFP pull-down experiments) facilitate biotinylation of individuals of the same protein in close proximity, thus the higher intensities compared to NS1-*wt*, which has more interaction partners.

We analyzed the proteins identified by the GFP pull-down or the BioID2 approach to determine biological processes that are potentially regulated by NS1-wt and NS-T585E. Amongst the identified biological processes, we focused on biological processes relevant for NS1 activity based on previous publications [2]. This did include viral protein processing biological processes, regulation of gene expression, regulation of the cell cycle, DNA damage repair, stress response, and regulation of the immune system. In addition, proteins were analyzed for their association with cellular compartments, such as the ribonucleoprotein complex, the mitochondrion, and the cytoskeleton.

Surprisingly, analysis of the GFP approach but not the BioID2 approach identified biological processes involved in DNA damage or regulation of the immune system. This might be explained by the C-terminal localization of BioID2 within the NS1-BioID2 fusion protein. The biotinylation radius of BioID2 is restricted to a recommended [56] radius of 20–30 nm. It is therefore possible that certain proteins localized near N-terminal domains might be missed.

As mentioned, NS1-T585E interacts with fewer proteins but seems to bind individuals of one protein more often. Proteins which show a much higher binding-fraction of NS1-T585E/mock compared to NS1-wt/mock include mainly proteins of the gene expression machinery RPL11 (component of the 60S ribosomal subunit), SRSF10 (RNA splicing), and TRA2B (mRNA splicing) as well as cycle progression and DNA replication (namely RFC1 large subunit of the replication factor C), PRIM2 (subunit of DNA primase), and CDK11B (cyclin dependent kinase11)). These findings are in line with the strong involvement of NS1 in the host-cell gene expression machinery and cell cycle progression as described previously. They also support the notion that NS1 mediated disruption of host-cell gene expression and cell cycle arrest partly contributes to the apoptotic effects of NS1 [2,10,57]. Noteworthy is Calmodulin1, which is bound only by NS1-T585E but not by NS1-wt. Calmodulin1 regulates a multitude of physiological processes like cell proliferation, programmed cell death and autophagy and has a major impact on the regulation of several specific cell cycle phases. It is also involved in processes required for tumor progression such as growth, tumor-associated angiogenesis and metastasis [58]. Another interesting protein is Apoptotic Chromatin Condensation Inducer 1 (ACIN1) which is about 7.4 times more abundant in the NS1-T585E-GFP compared to the NS1-wt-GFP pulldown and which also shows a different phosphorylation pattern after both NS1-wt and NS1-T585E expression (see section below). Taken together, these findings may provide an explanation for the stronger cytotoxic effect of NS1-T585E.

### 4.4. Impact of NS1 on Phosphorylation Patterns

NS1-wt overexpression led to a significantly altered phosphorylation pattern of four proteins: Apoptotic Chromatin Condensation Inducer 1 (ACIN1) is known to induce apoptotic chromatin condensation after activation by caspase-3 [59]. Residue T254 of ACIN1 showed a higher phosphorylation after NS1-T585E and NS1-wt expression compared to mock transfection. This indicates a difference in ACIN1 regulation upon both, NS1-T585E and NS1-wt expression. The lower phosphorylation of residue T254 after NS1-T585E compared to NS1-wt expression might indicate a difference in the regulation of ACIN1. However, as phosphorylation of target proteins can be a highly dynamic process, which may lead to a variety of effects as gain and loss of functions, no conclusion about what exactly the outcome of this difference is can be drawn [38]. ACIN1 is about 7.4 times more abundant in the NS1-T585E-GFP compared to the NS1-wt-GFP pull-down. The protein is activated by caspase 3 upon which it induces apoptotic chromatin condensation [59]. Caspase 3 activation upon NS1-wt expression has previously been described [9]. The direct interaction of NS1-T585E and ACIN1 which is much more pronounced than the interaction of NS1-wt and ACIN1, together with the altered ACIN1 phosphorylation pattern upon NS1 (*wt* and *mutant*) expression might lead to an explanation on how NS1-T585E leads to a much stronger cytotoxic effect.

Phosphorylation of Small Glutamine Rich Tetratricopeptide Repeat Co-Chaperone Alpha (SGTA) is in line with the observed upregulation upon NS1 overexpression and the previously described association with NS1-wt [54].

HIRA Interacting Protein 3 (HIRIP3) is suggested to play a role in histone and chromatin metabolism [60]. HIRIP3 has been proposed as a substrate of CK2 [60]. Interference of NS1 with CK2 signaling has been shown to be important for the cytopathic effects of NS1 [44]. HIRIP3 has also been proposed to be involved in chromatin metabolism and to be dephosphorylated and excluded from the nucleus during chromatin condensation. This could be another indication of an NS1 induced phosphorylation and activation of ACIN1 [61].

The function of AMMECR Nuclear Protein 1 (AMMECR1) has been shown to inhibit apoptosis and to promote cell cycle progression in cancer cells [62]. Its regulation by NS1 could also be linked to the apoptotic effects and cell cycle arrest upon NS1 expression in cancer cells.

We believe that phosphorylations are necessary to expose interfaces for interactions with cell proteins. However, additional features such as the subcellular distribution of the viral protein into microdomains in the cytoplasm are crucial to bring the effector to the target. This is very well controlled upon virus infection rearranging, for instance, the cytoskeleton and in consequence membrane structure [63].

Interestingly, we could show that NS1-wt directly or indirectly binds alpha and beta tubulins. Phosphorylation of tubulins in cells after NS1-expression has been described and a link to cytoskeleton disruption has been proposed [63]. In the case of NS1-T585E, however, close proximity, but not direct or indirect binding to tubulins could be demonstrated.

### 4.5. Additional NS1 Interaction Partners and Pathways

In the present work, NS1 interaction partners were identified and discussed based on statistical evaluation of proteomics data. They were selected according to two criteria: First, statistical relevance, i.e., a q value of ≤0.05 compared to mock transfected control cells. Second, statistical association with a relevant biological process. It should be noted, however, that purely statistical criteria might fail to identify important NS1 partners or biological processes. Subtle upstream modulation of signaling pathways, for example, can have a profound impact on downstream biological processes. Based on literature data, we have therefore reanalyzed the available dataset and manually selected proteins of interest.

Focusing on pathways, identified to contribute to successful infection, progeny particle production and spreading, we found a number of these cell proteins being connected to NS1 after transfection of Hep3B cells. For viral DNA amplification, a reconstituted in-vitro replication system identified, besides polymerase, RPA, RFC, PCNA being essential to drive leading strand synthesis driven by NS1’s helicase activity [64]. In addition to these basic replication factors, other NS1-interaction partners identified here, were shown to accumulate in PV-induced replication (APAR-) bodies and others functioning in the DNA-damage response/repair (H2AFY/MCM4) and cell cycle control (cdk11B), processes that were described to contribute to efficient DNA replication and progeny particle production [57,65,66]. Besides strongly validating our results, these findings lead us to suggest that additional interaction partners might provide new insights into the functioning of additional parvoviral mechanisms, such as efficient mRNA processing/splicing regulation for which additional candidates to hnRNPs were identified [67]. Together with the finding of tRNA ligases, potentially facilitating an amber-read-through to generate the proposed non-structural protein NS3, this might enable us to pinpoint the potential functioning of splicing regulation for the generation of viral proteins, particularly in the presence of an innate immune-response (interaction with translation initiation factors).

Interestingly, besides expected NS1-interactions facilitating progeny particle production, there are a number of cellular factors that could serve therapeutic applications using this viral protein as an oncotoxin. Besides factors involved in regulated death-pathways (i.e., interference with the energy metabolism, apoptosis and autophagy) there are more (parvo)viral specific pathways leading to cell disturbances and release of intracellular components modulating cytoskeleton dynamics and exocytosis. The latter is of particular interest, since these factors are indicative for the potential NS1-induced release of DAMPs and PAMPs, in order to induce an anti-tumor immune-response. In addition to chaperones, which were shown to play a significant role for the induction of anti-tumor immune responses, we identified a number of TAAs interacting with NS1 (i.e., TPT1, NUMA1, SPRYD4, SND1, and GNL3). If such polypeptides are released together with NS1, this might significantly contribute to a potential therapy. Indeed, during a first clinical trial using parvovirus H-1PV, immune-activation could be confirmed, including a response against the non-structural protein NS1 [19].

## 5. Conclusions

Our findings provide an insight into the manifold, multimodal interplay of the NS1 protein and the proteome of NS1-sensitive cancer cells. These findings are instrumental to better understand the tumor-cell specificity and mode of action of NS1. The increased cytotoxic effect of NS1-T585E in NS1-wt-responsive cells, but not in NS1-wt-unresponsive cells, makes NS1-T585E an interesting candidate for the design of safe and efficient NS1-based cancer treatment strategies. The insights on H1-PV—host-cell interactions on a molecular level, provided by this work, will pave the way for further experiments including the development of novel treatment strategies. Such therapeutic approaches might be a combination of viral vector-mediated high-efficiency transduction, stimulation of death pathways, and anti-tumor immune stimulation. Proof-of-concept studies will be carried out using in vivo xenograft murine tumor models.

## Figures and Tables

**Table 1:** Plasmids and Primers used for cloning and mutant design.

Plasmid	PCR Template	Primers	Expressed Protein
pcDNA3.1+	original plasmid (Invitrogen)	-	no protein expression
pcDNA3.1—NS1	Witzigmann et al. [3]	-	NS1-wt
pcDNA3.1—NS1 (K85Q)	pcDNA3.1—NS1	p1, p7; p6, p2	NS1-K85Q
pcDNA3.1—NS1 (K257Q)	pcDNA3.1—NS1	p1, p9; p8, p2	NS1-K257Q
pcDNA3.1—NS1 (S283E)	pcDNA3.1—NS1	p1, p11; p10, p2	NS1-S283E
pcDNA3.1—NS1 (T435E)	pcDNA3.1—NS1	p1, p13; p12, p2	NS1-T435E
pcDNA3.1—NS1 (S473E)	pcDNA3.1—NS1	p1, 15; p14, p2	NS1-S473E
pcDNA3.1—NS1 (T585A)	pcDNA3.1—NS1	p1, 17; p16, p2	NS1-T585A
pcDNA3.1—NS1 (T585E)	pcDNA3.1—NS1	p1, p19; p18, p2	NS1-T585E
pcDNA3.1—NS1 (d114)	pcDNA3.1—NS1	p1, p20; p21, p2	NS1-d114
pTag-GFP-N	original plasmid (Evrogen)	-	GFP
pTag-NS1-GFP	Witzigmann et al. [3]	-	NS1-wt-GFP
pTag-NS1(T585E)-GFP	pcDNA3.1—NS1 (T585E)	p1, p3	NS1-T585E-GFP
MCS-BioID2-HA	original plasmid (Addgene)	-	no protein expression
pBioID2	MCS-BioID2-HA	p22, p23	BioID2
pNS1-BioID2	pcDNA3.1—NS1	p4, p5	NS1-wt-BioID2
pNS1(T585E)-BioID2	(T585E)	p4, p5	NS1-T585E-BioID2

## Data Availability

Data generated or analysed during this study can be found within the published article and its Supplementary Files. All raw data files and statistical values used for analysis are provided in the repository zenodo.org (https://doi.org/10.5281/zenodo.6423418, accessed on 7 April 2022). Proteomics data are also available via ProteomeXchange with identifier PXD036350.

## Supplementary Materials

The following supporting information can be downloaded at: https://www.mdpi.com/article/10.3390/v15010209/s1, Table S1: List of PCR primers, Figure S1: Determination of reactive oxygen species (ROS) production, Figure S2: Determination of reactive oxygen species (ROS) production, Figure S3: Viability of transfected primary mouse hepatocytes, Figure S4: Volcano plot depicting the changes in the proteome after NS1-wt and NS1-T585E expression, Figure S5: Volcano plot depicting the changes of protein phosphorylation after NS1-wt and NS1-T585E expression.
