# Supplementary material for: Design and Characterization of Mutated Variants of the Oncotoxic Parvoviral Protein NS1
Source: Viruses. 2023 Jan 11;15(1):209. doi: 10.3390/v15010209 (PMC9866090; doi:10.3390/v15010209)
Supplement: Supplementary file 1 [file viruses-15-00209-s001.zip › viruses-2107811-supplementary.pdf]

Supplementary Material

Table S1: List of PCR primers. Mutated amino acid codons are marked in bold.

#	Plasmid	Primer sequence
p1	BamHI-NS1-fwd	ATTGGATCCGCGATGGCTGGAAACGCTTACTCC
p2	NotI-NS1-rev	TATTAATGCGGCCGCTTAGTCCAAGGTCAGCTCCTCG
p3	BamHI-NS1-rev	AATGGATCCGCGTCCAAGGTCAGCTCCTCG
p4	AgeI-NS1-fwd	AGCACCGGTGACATGGCTGGAAACGCTTACTCC
p5	AgeI-NS1-rev2	AATACCGGTTGTCCAAGGTCAGCTCCTCG
p6	NS1-K85Q-fwd	AGCGCAGTGGATGACATGACCAAA**CAG**CAAGTATTTATT
p7	NS1-K85Q-rev	AATAAATACTTG**CTG**TTTGGTCATGTCATCCACTGCGCT
p8	NS1-K257Q-fwd	CTGTATACTGATGAGATG**CAA**CCAGAAACGGTCGAGACC
p9	NS1-K257Q-rev	TCTCGACCGTTTCTGG**TTG**CATCTCATCAGTATACAG
p10	NS1-S283E-fwd	CAAACTAGAAAGGAGGTC**GAG**ATTAAAACCACACTCAAA
p11	NS1-S283E-rev	TTTGAGTGTGGTTTTAAT**CTC**GACCTCCTTTCTAGTTTG
p12	NS1-T435E-fwd	TTTCCATTTAATGACTGT**GAG**AACAAAAACTTGATTTGG
p13	NS1-T435E-rev	CCAAATCAAGTTTTTGTT**CTC**ACAGTCATTAAATGGAAA
p14	NS1-S473E-fwd	GATCAAAAAGGAAAAGGC**GAG**AAACAGATTGAACCAACAC
P15	NS1-S473E-rev	GTGTTGGTTCAATCTGTTT**CTC**GCCTTTTCCTTTTTGATC
p16	NS1-T585A-fwd	CGCTCACCATCTCTG**GCT**CCGAGAAGTACGCCTCTCAGC

p17	NS1-T585A-rev	GCTGAGAGGCGTACTTCTCGG**AGC**CAGAGATGGTGAGC
p18	NS1-T585E-fwd	CTCACCATCTCTG**GAG**CCGAGAAGTACGCCT
p19	NS1-T585E-rev	AGGCGTACTTCTCGG**CTC**CAGAGATGGTGAG
p20	NS1-d114-rev	CAGCCTCCCCAGTGTTTTGGCTCGGAGTCAGAGATGGTGA
p21	NS1-d114-fwd	TCACCATCTCTGACTCCGAGCCAAAACACTGGGGAGGCTG
p22	AgeI-ATG-BioID2-fwd	ATACCAACCGGTGAC**ATG**TTCAAGAACCTGATCTGGCTGA
p23	HindIII-stop-HA-rev	TATACGTAAGCTTCTATGCGTAATCCGGTACATCGT

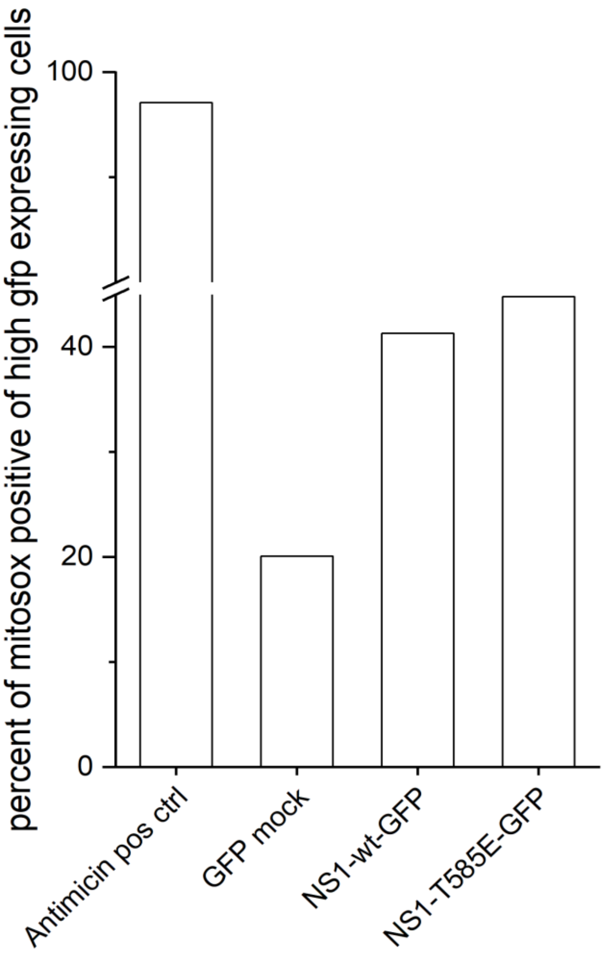

Figure S1: Determination of reactive oxygen species (ROS) production. Intracellular ROS levels were determined in responder Hep3B cells using the MitoSox assay (ThermoFischer) 72h post transfection. Cells were transfected with NS1-wt GFP or NS1-T585E GFP. Antimicin A treated cellswere uses as a positive control. GFP expressing cells were used as a negative control. >1400 high GFP expressing cells per group were counted.

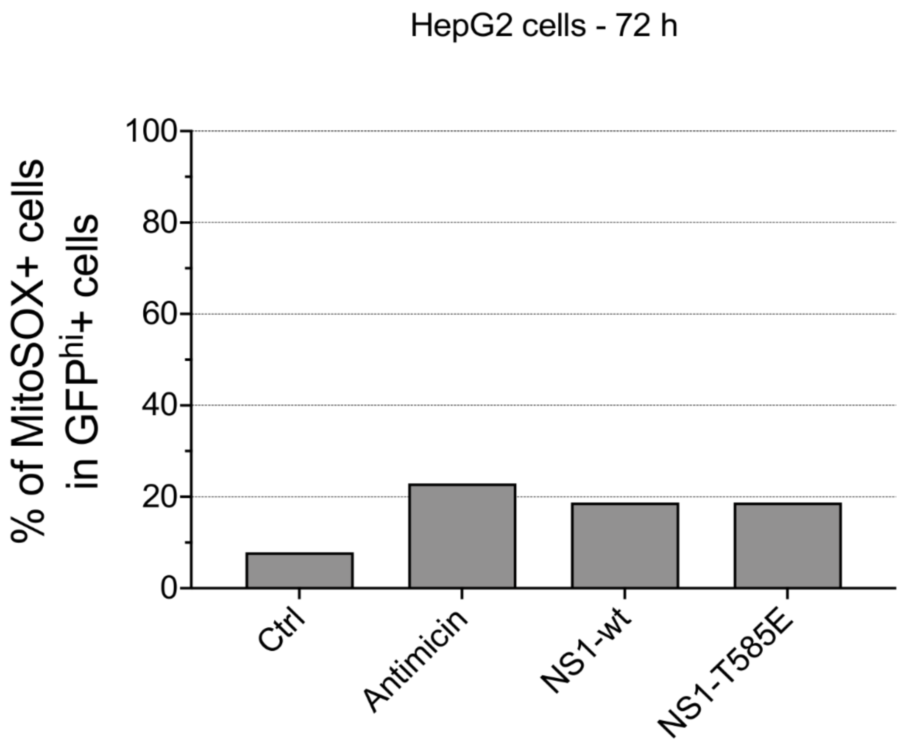

Figure S2: Determination of reactive oxygen species (ROS) production. Intracellular ROS levels were determined in non-responder HepG2 cells using the MitoSox assay (ThermoFischer) 72h post transfection. Cells were transfected with NS1-wt GFP or NS1-T585E GFP. Antimicin A treated cellswere uses as a positive control. GFP expressing cells were used as a negative control. >1400 high GFP expressing cells per group were counted.

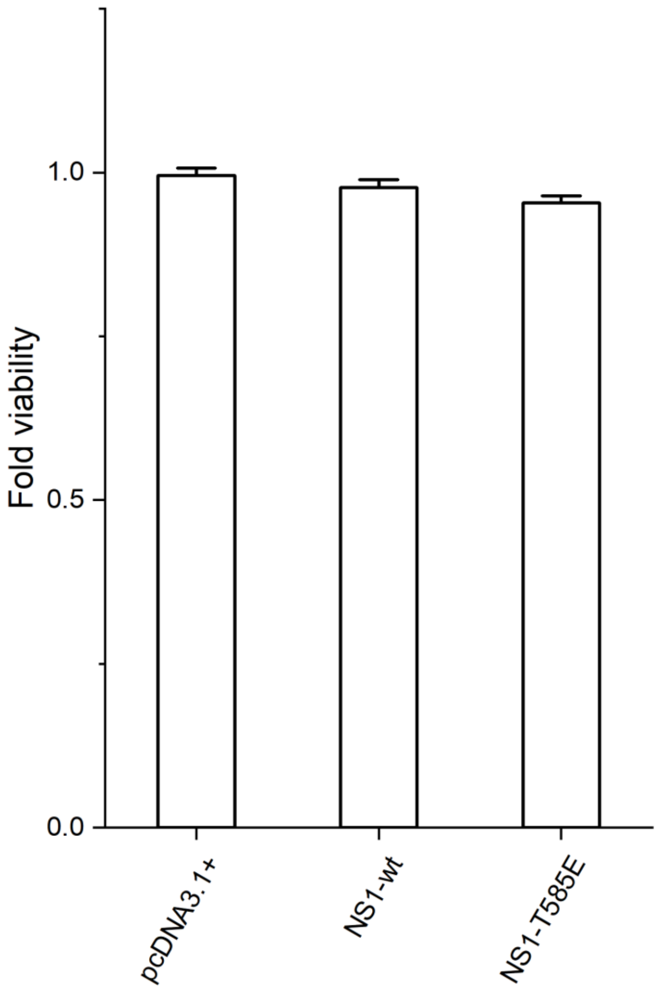

Figure S3: Viability of transfected primary mouse hepatocytes. Viability determined by the MTT assays of non-responder primary hepatocytes transfected with empty pcDNA3.1+ vector (mock), pcDNA3.1-NS1-wt, or with pcDNA3.1-NS1-mutant. Relative viability was determined 72h post transfection and normalized to pcDNA3.1+ (mock) transfection. Values are means ± SEM (n = 10 measurements).

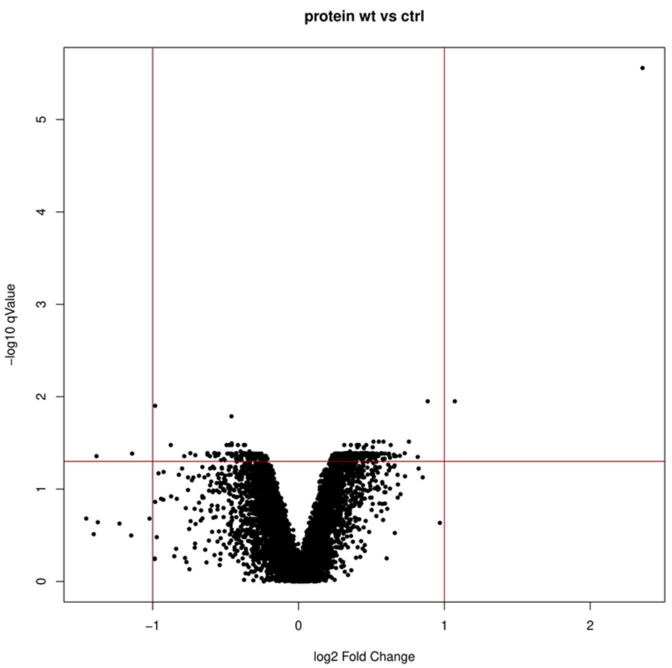

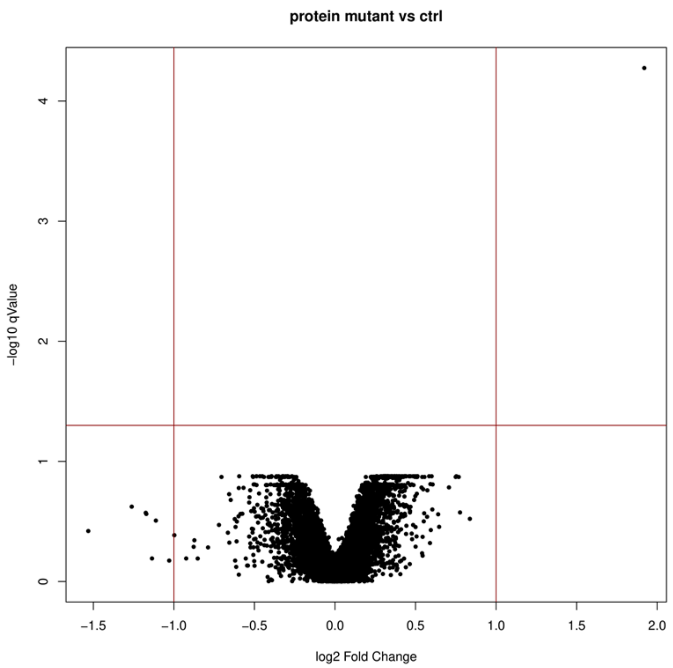

Figure S4: Volcano plot depicting the changes in the proteome after NS1-wt (left) and NS1-T585E (right) expression. Log(2) of ratios of protein levels after NS1 (wt, left or mutant T585E, right) and empty plasmid (mock) transfection of Hep3B cells (x-axis) blotted against the neg. log(10) of corresponding q-values (y-axis).

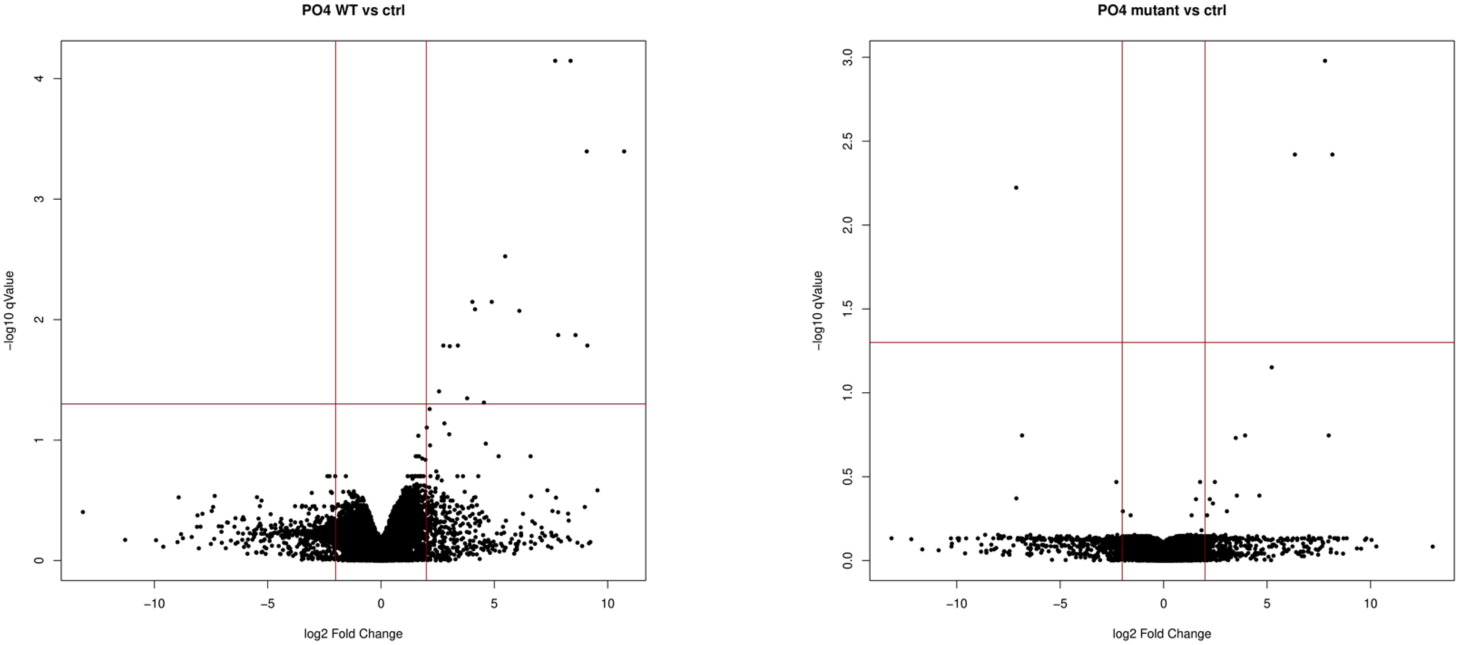

Figure S5: Volcano plot depicting the changes of protein phosphorylation after NS1-wt (left) and NS1-T585E (right) expression. Log(2) of ratios of identified phosphorylated proteins after NS1 (wt, left or mutant T585E, right) and empty plasmid (mock) transfection of Hep3B cells (x-axis) blotted against the neg. log(10) of corresponding q-values (y-axis).
